# Formation and Dissociation of Phosphorylated Peptide Radical Cations

**DOI:** 10.1007/s13361-012-0479-7

**Published:** 2012-09-12

**Authors:** Ricky P. W. Kong, Quan Quan, Qiang Hao, Cheuk-Kuen Lai, Chi-Kit Siu, Ivan K. Chu

**Affiliations:** 1 Department of Chemistry, University of Hong Kong, Hong Kong, China; 2 Department of Biology and Chemistry, City University of Hong Kong, Hong Kong, China

**Keywords:** Formation, Dissociation, Collision-induced dissociation, Phosphorylated peptide radical cations, DFT calculation

## Abstract

In this study, we generated phosphoserine- and phosphothreonine-containing peptide radical cations through low-energy collision-induced dissociation (CID) of the ternary metal–ligand phosphorylated peptide complexes [Cu^II^(terpy)_*p*_M]^·2+^ and [Co^III^(salen)_*p*_M]^·+^ [_*p*_M: phosphorylated angiotensin III derivative; terpy: 2,2':6',2''-terpyridine; salen: *N*,*N* '-ethylenebis(salicylideneiminato)]. Subsequent CID of the phosphorylated peptide radical cations (_*p*_M^·+^) revealed fascinating gas-phase radical chemistry, yielding (1) charge-directed b- and y-type product ions, (2) radical-driven product ions through cleavages of peptide backbones and side chains, and (3) different degrees of formation of [M – H_3_PO_4_]^·+^ species through phosphate ester bond cleavage. The CID spectra of the _*p*_M^·+^ species and their non-phosphorylated analogues featured fragment ions of similar sequence, suggesting that the phosphoryl group did not play a significant role in the fragmentation of the peptide backbone or side chain. The extent of neutral H_3_PO_4_ loss was influenced by the peptide sequence and the initial sites of the charge and radical. A preliminary density functional theory study, at the B3LYP 6-311++G(d,p) level of theory, of the neutral loss of H_3_PO_4_ from a prototypical model—*N*-acetylphosphorylserine methylamide—revealed several factors governing the elimination of neutral phosphoryl groups through charge- and radical-induced mechanisms.

**Electronic supplementary material:**

The online version of this article (doi:10.1007/s13361-012-0479-7) contains supplementary material, which is available to authorized users.

## Introduction

Protein post-translational modifications (PTMs) are essential processes in the regulation of cellular events because they are necessary steps toward rendering the functionality of a protein [1]. Phosphorylation is a common PTM; in many proteins, the hydroxyl (–OH) group of the side chain of a tyrosine, serine, or threonine residue is modified with an inorganic phosphoryl [–OPO(OH)_2_] group to activate or deactivate cell signaling processes [1]. The exact phosphorylation site(s) of proteins cannot be predicted directly from known genomes; deciphering the sequences of phosphorylated peptides is, therefore, a necessary step toward understanding the functions of the proteins [2].

Mass spectrometry (MS)-based identification of phosphopeptides involves analysis of fragmentation patterns to determine the peptide sequence and the number and exact positions of the phosphorylation sites. Despite recent advances in MS-based proteomics, determining the exact modification sites of phosphorylated peptides remains challenging [3–6], partly because deciphering phosphopeptide sequences from fragmentation patterns requires an understanding of the remarkable range of chemistry that can occur during the dissociation process. Phosphoester [C–OPO(OH)_2_] bonds are labile during collision-induced dissociation (CID), potentially resulting in facile elimination of the covalently bonded phosphoryl moieties, through the loss of neutral inorganic phosphates, even under low-energy CID conditions. In contrast to the traditional sequencing approaches based on CID of even-electron protonated peptides [M + *n*H]^*n*+^ [7, 8], the gas-phase dissociations of odd-electron peptides [M + *n*H]^·(*n*–1)+^, generated through electron capture dissociation (ECD) or electron transfer dissociation (ETD), can be used to determine the sites of PTMs, partly because they involve specific cleavages along the peptide backbone under controllable conditions, in some cases retaining the labile modified groups [9, 10]. Alternative approaches for the formation of radical peptide cations include multiphoton laser desorption ionization of peptides featuring aromatic chromophores [11–13], CID of peptides derivatized with a free-radical initiator or labile radical precursor [14, 15], laser photolysis of peptides containing photolabile tags [16, 17], and one-electron oxidative dissociation of ternary metallopeptide complexes induced by CID [18–21]. The latter method, with judicious choice of the transition metal and ligand for the metal complex, has allowed us to prepare a variety of both cationic and anionic radical peptides (M^·+^, [M + H]^·2+^, [M – 2H]^·–^) within various commercial tandem mass spectrometers, including triple-quadrupole, three-dimensional or linear quadrupole ion trap, and hybrid quadrupole time-of-flight tandem mass spectrometers, equipped with an electrospray ionization (ESI) source [22–26].

In this study, we extended the established metal–ligand complex method to the generation of _*p*_M^·+^ species, opening up a fruitful exploration of their chemistry. To the best of our knowledge, this approach has not been employed previously for the generation of _*p*_M^·+^ radical cations [22, 23, 26]. Here, we synthesized a series of _*p*_M^·+^ radical cations in situ within a quadrupole ion trap mass spectrometer. The dissociation of these novel _*p*_M^·+^ species is substantially different from that of their protonated counterparts; understanding their dissociation chemistry is, therefore, a significant and important step toward dissecting the fundamental factors governing the extent of neutral H_3_PO_4_ loss.

## Experimental

### Materials

All chemicals were obtained commercially (Aldrich and Sigma, St. Louis, MO, USA; Bachem, King of Prussia, PA). Fmoc-protected amino acids and Wang resin were purchased from Advanced ChemTech (Louisville, KY, USA). The phosphopeptides were synthesized in-house using standard Fmoc synthesis strategies, as described previously [27], and used without further purification. Cu(II)(terpy)(NO_3_)_2_ (terpy: 2,2';6',2''-terpyridine) and [Co(III)(salen)]Cl [salen: *N*,*N* '-ethylenebis(salicylideneiminato)] complexes were synthesized according to previously reported procedures [28, 29].

### Mass Spectrometry

All experiments were conducted using a quadrupole ion trap mass spectrometer (Finnigan LCQ, ThermoFinnigan, San Jose, CA, USA). Samples typically comprised 600 μM metal complex and 50 μM peptide in a water/methanol (50:50) solution. They were introduced into the mass spectrometer through direct infusion (2.0 μL/min) via the electrospray ionization (ESI) source. The injection and activation times for CID in the ion trap were 200 and 30 ms, respectively; the amplitude of the excitation was optimized for each experiment.

### Computational Methods

The geometric structures of the model systems—*N*-acetylphosphorylserine methylamide analogues—were optimized in the framework of density functional theory (DFT) at the unrestricted B3LYP/6-311++G(d,p) level, as implemented in the Gaussian 03 quantum chemistry package [30]. Local minima and transition structures were identified with zero and one imaginary vibrational frequency, respectively, as obtained from harmonic frequency analyses. The local minima associated with each transition state structure were also confirmed through calculations of the intrinsic reaction coordinates.

## Results and Discussion

### Formation of Phosphopeptide Radical Cations (_p_M^·+^)

**Figure 1:**
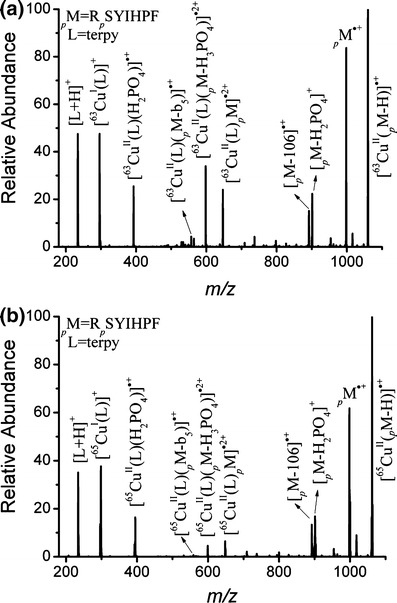
CID spectra of **(a)** [^63^Cu^II^(terpy)_*p*_M]^·2+^ and **(b)** [^65^Cu^II^(terpy)_*p*_M]^·2+^ (_*p*_M = R_p_SYIHPF)

We first investigated the generation of phosphopeptide radical cations (_*p*_M^·+^) through CID of copper(II)–ligand–peptide complexes. Figure 1a displays the CID spectrum of [^63^Cu^II^(L)(_*p*_M)]^·2+^, where L is terpy and _*p*_M is R_*p*_SYIHPF, an angiotensin III derivative modified with a phosphorylated serine residue (_*p*_S). The spectrum features abundant signals at *m*/*z* 998.2, assigned to _*p*_M^·+^, and *m*/*z* 296.1, assigned to the complementary reduced product ion [^63^Cu^I^(L)]^+^. We confirmed these spectral assignments by comparing the CID spectra of [^63^Cu^II^(L)(_*p*_M)]^·2+^ with that of its isotope analogue [^65^Cu^II^(L)(_*p*_M)]^·2+^ (Figure 1b). The signals of the singly and doubly charged copper-containing ions in the spectrum of [^65^Cu^II^(L)(_*p*_M)]^·2+^ are shifted by *m*/*z* +2 and +1, respectively, relative to the corresponding signals in the spectrum of [^63^Cu^II^(L)(_*p*_M)]^·2+^. The corresponding peaks for _*p*_M^·+^ and [^65^Cu^I^(L)]^+^ are centered at *m*/*z* 998.3 and 298.1, respectively; the signal for _*p*_M^·+^ is not shifted, whereas that for [^65^Cu^I^(L)]^+^ is shifted by *m*/*z* +2. Next, we examined the CID spectra of copper(II)–terpy complexes of a series of angiotensin phosphopeptide analogues featuring amino acid residues possessing various ionization energies and proton affinities; the behavior of the [Cu^II^(terpy)]^·2+^-mediated phosphorylated peptide radical cations (Table 1) was generally in accordance with that of their non-phosphorylated counterparts [26, 31], _ENREF_30 despite minor variations in the relative abundances of fragment ions and the types of observable low-abundance product ions.

**Table 1:** Metal complexes and relative abundances of _*p*_M^·+^ species, together with relative abundances for the two most competitive fragments. The percentage abundances of the neutral losses of phosphoric acid from _*p*_M^·+^ ([_*p*_M – H_3_PO_4_]^·+^) and [_*p*_M + H]^+^ ([_*p*_M + H – H_3_PO_4_]^+^) are also listed

Phosphorylated peptide(_*p*_M)	Metal ligand complex	% _*p*_M^·+^from metal ligand complex	Two most competitive fragmentations from metal ligand peptide (relative abundance %)	%H_3_PO_4_ loss from _*p*_M^·+^	%H_3_PO_4_ loss from [_*p*_M + H]^+^
G_*p*_SGIHPY	Co^III^(salen)	100	[_*p*_M + H]^+^(42), [Co^III^(salen)(_*p*_M – H_3_PO_4_)]^+^(21)	80.7	97.7
GVGIH_*p*_SY	Co^III^(salen)	76	[_*p*_M + H]^+^(100), [_*p*_M – CO_2_]^·+^(31)	14.9	53.6
RVYIH_*p*_SF	Co^III^(salen)	73	[_*p*_M + H]^+^(100), [_*p*_M – COOH] ^+^(45)	39.4	61.2^a^
RVGIH_*p*_SY	Co^III^(salen)	42	[Co^III^(salen)(_*p*_M – H_3_PO_4_)]^+^(100), [Co^III^(salen)(_*p*_M – NH_3_)]^+^(45)	59.5	86.1
GVYIH_*p*_SF	Co^III^(salen)	20	[_*p*_M + H]^+^(100), [Co^III^(salen)(_*p*_M – H_3_PO_4_)]^+^(20)	19.3	44.8
R_*p*_SGIHPY	Co^III^(salen)	100	[_*p*_M – CO_2_]^·+^(40), [_*p*_M + H]^+^(39)	54.6	73.7
	Cu^II^(terpy)	48	[Cu^II^(_*p*_M – H)]^+^(100), [Cu^I^(terpy)]^+^(43)	37.6	
R_*p*_SYIHPF	Co^III^(salen)	100	[Co^III^(salen)b_5_]^+^(17), [_*p*_M – CO_2_]^·+^(16)	79.4	87.6
	Cu^II^(terpy)	84	[Cu^II^(_*p*_M – H)]^+^(100), [Cu^I^(terpy)]^+^(48)	61.7	
G_*p*_SYIHPF	Co^III^(salen)	72	[_*p*_M + H]^+^(100), [Co^III^(salen)(_*p*_M – H_3_PO_4_)](34)	92.7	96.7
	Cu^II^(terpy)	82	[_*p*_M – H_2_PO_4_]^+^(100), [Cu^II^(terpy)H_2_PO_4_]^+^(87)	93.1	
RGL_*p*_SYG	Co^III^(salen)	76	[_*p*_M + H]^+^(100), [_*p*_M – 106]^·+^(30)	29.0	90.0
	Cu^II^(terpy)	100	[Cu^I^(terpy)]^+^(74), [_*p*_M – 106]^·+^(53)	27.3	
GGL_*p*_SYG	Cu^II^(terpy)	37	[Cu^II^(terpy)H_2_PO_4_]^+^(100), [_*p*_M – H_2_PO_4_]^+^(82)	8.0	43.4
RGG_*p*_SY	Cu^II^(terpy)	100	[Cu^I^(terpy)]^+^(98), [_*p*_M – 106]^·+^(71)	40.3	85.7
RGG_*p*_SW	Cu^II^(terpy)	1	[Cu^II^(terpy)H_2_PO_4_]^+^(100), [_*p*_M – H_2_PO_4_]^+^ (82)	19.6	84.2
GG_*p*_SY	Cu^II^(terpy)	18	[Cu^II^(terpy)H_2_PO_4_]^+^(100), [_*p*_M – H_2_PO_4_]^+^(84)	7.1	76.3
GG_*p*_SW	Cu^II^(terpy)	44	[Cu^II^(terpy)H_2_PO_4_]^+^(100), [_*p*_M – H_2_PO_4_]^+^ (64)	1.7	75.2

^a^Dividing the integrated area of the [_*p*_M – H_3_PO_4_]^·+^ or [_*p*_M + H – H_3_PO_4_]^+^ peak by the total area of all product ion peaks with peak heights greater than 0.5 % of the maximum peak height in the corresponding peptide radical cation or protonated peptide spectra

^b^The most predominant further fragment y_6_^*+^ ion is also taken into account

**Scheme 1:**
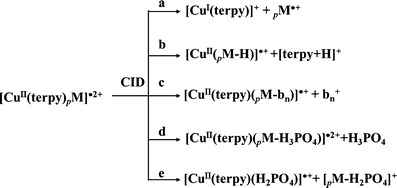
Collision-induced dissociation pathway of [Cu^II^(terpy)_*p*_M]^·2+^ complex, where _*p*_M = phosphoserine or phosphothreonine peptide

Scheme 1 summarizes the elucidated CID pathways for [Cu^II^(L)(_*p*_M)]^·2+^. Scheme 1a displays the reaction resulting in the formation of _*p*_M^·+^ and the reduction of the copper(II)–ligand complex to [^63^Cu^I^(L)]^+^, as evidenced by the aforementioned signals at *m*/*z* 998.2 and 296.1. Other pathways occurring during the CID of [Cu^II^(L)(_*p*_M)]^·2+^ include proton transfer from the phosphopeptide to the ligand (Scheme 1b), phosphopeptide fragmentation (Scheme 1c), neutral loss of H_3_PO_4_ (Scheme 1d), and heterolytic cleavage of the phosphoester bond (Scheme 1e). Our CID spectra of [^63^Cu^II^(L)(_*p*_M)]^·2+^ reveal evidence for each of these pathways (Figure 1a). Proton transfer from the phosphopeptide to the ligand resulted in the formation of [^63^Cu^II^(L)(_*p*_M – H)]^·+^ and [L + H]^+^ ions at *m*/*z* 1060.0 and 234.1, respectively. Phosphopeptide fragmentation was evidenced by the appearance of weaker signals for [^63^Cu^II^(L)(_*p*_M – b_5_)]^·+^ and b_5_^+^ species, at *m*/*z* 557.0 and 737.2, respectively. We also identified a channel for neutral H_3_PO_4_ loss yielding the [^63^Cu^II^(L)(_*p*_M – H_3_PO_4_)]^·2+^ fragment at *m*/*z* 598.2. Finally, signals for the charge-separated product ion pair [^63^Cu^II^(L)(H_2_PO_4_)]^·+^ and [_*p*_M – H_2_PO_4_]^+^, at *m*/*z* 392.9 and 901.2, respectively, provided evidence for heterolytic cleavage of the phosphoester bond.

Extended preliminary studies revealed the applicability of performing the electron transfer reaction using various triply charged transition metals, such as [Co^III^(L)_*p*_M]^·+^ (L = salen). In general, the primary dissociation pathways of the phosphopeptide complexes of [Cu^II^(L)_*p*_M]^·2+^ (Scheme 1) and [Co^III^(L)_*p*_M]^·+^ (Supplementary–Scheme S1) resemble those of the analogous non-phosphorylated peptide metal complexes[20, 26, 31], except for the additional channels related to H_3_PO_4_ loss. Our present results indicate that it is indeed possible to extend the one-electron oxidative dissociation of metal–peptide complexes to the gas-phase syntheses of novel cationic phosphorylated radical peptides.

### Dissociation of Phosphopeptide Radical Cations

**Figure 2:**
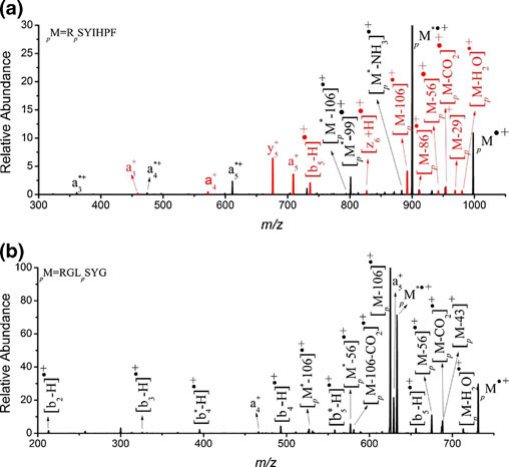
CID spectra of **(a)** [R_*p*_SYIHPF]^·+^ (ions in common with RSYIHPF are marked in red) and **(b)** [RGL_*p*_SYG]^·+^, the asterisk (*) denotes the loss of H_3_PO_4_

**Figure 3:**
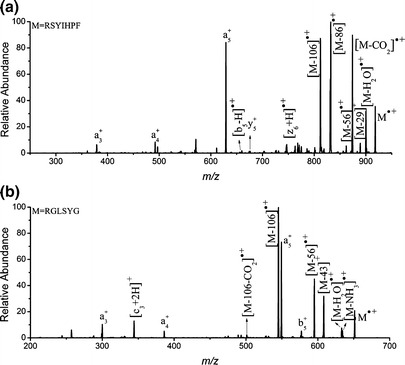
CID spectra of **(a)** [RSYIHPF]^·+^ and **(b)** [RGLSYG]^·+^

Upon successful generation of phosphoserine- or phosphothreonine-containing peptide radical cations, we studied the gas-phase dissociations of phosphorylated-angiotensin III derivatives to elucidate the fundamental factors governing their competitive fragmentation mechanisms, in particular, their phosphate ester bond cleavages. The CID spectrum of [R_*p*_SYIHPF]^·+^ features an intense signal at *m*/*z* 900.2, which we assign to the fragment ion obtained after neutral loss of H_3_PO_4_ (98 Da), denoted _*p*_M^*·+^ (Figure 2a). We also observed additional CID pathways for [R_*p*_SYIHPF]^·+^, namely radical-driven side-chain cleavages, cleavage of peptide backbones, neutral losses, and multiple neutral losses. First, a signal for radical-driven side-chain cleavage of tyrosine appeared at *m*/*z* 892.2 ([_*p*_M – 106]^·+^, from the loss of CH_2_ = C_6_H_4_ = O); cleavage of isoleucine was observed at *m*/*z* 969.2 ([_*p*_M – 29]^+^, the loss of ^·^CH_2_CH_3_) and 942.1 ([_*p*_M – 56]^·+^, the loss of CH_3_CH = CHCH_3_); and cleavage of arginine was observed at *m*/*z* 912.1 {[_*p*_M – 86]^+^, the loss of ^·^CH_2_CH_2_NH(C = NH)NH_2_}. Second, cleavage of the peptide backbone with or without phosphorylation gave rise to peaks assigned to a_n_^*+^, a_n_^+^, y_n_^+^, [b_n_ – H]^·+^, and [z_n_ + H]^·+^ species, where an asterisk (*) denotes loss of H_3_PO_4_. Third, neutral losses of H_2_O and CO_2_ resulted in signals at *m*/*z* 980.1 and 954.1, respectively. Finally, we observed peaks due to multiple neutral losses at *m*/*z* 801.2 {[_*p*_M^*^ – 99]^·+^, the losses of H_3_PO_4_ and the arginine side chain CH_2_ = CHCH_2_NH(C = NH)NH_2_} and 883.3 ([_*p*_M^*^ – 17]^·+^, the losses of H_3_PO_4_ and NH_3_). To confirm our spectral assignments for [R_*p*_SYIHPF]^·+^ (Figure 2a), we acquired CID spectra of the radical cations of two phosphorylated peptide analogs, [R_*p*_TYIHPF]^·+^ and [R_*p*_SYIHPL]^·+^ (i.e., by replacing the second N-terminus phosphoserine and first C-terminus phenylalanine residues with phosphothreonine and leucine residues, respectively). The CID spectrum of the phosphothreonine radical cation [R_*p*_TYIHPF]^·+^ reveals (Figure S1 **–**Supplementary) a fragmentation pattern almost identical to that of the corresponding phosphoserine radical cation ([R_*p*_SYIHPF]^·+^). Because of the extra methyl substituent on the β-carbon atom of the threonine residue, the N-terminus fragment ions (a_n_^+^, a_n_^*+^, [b_n_ – H]^·+^, [b_n_^*^ – H]^·+^) in the spectrum of the phosphothreonine are mass-shifted by *m*/*z* +14, whereas the C-terminus fragment ions (y_n_^+^, [z_n_ + H]^·+^) are not shifted relative to the spectrum of phosphoserine. Similarly, we confirmed our C-terminus spectral assignments through analysis of the phosphoserine peptide [R_*p*_SYIHPL]^·+^, in which a phenylalanine residue occupies the C-terminus. When we compare the CID spectra of these radical cations ([R_*p*_SYIHPF]^·+^ in Figure 2a and [R_*p*_SYIHPL]^·+^ in Figure S2 **–**Supplementary), the peaks due to C-terminus fragments (y_n_^+^, [z_n_ + H]^·+^) are mass-shifted by *m*/*z* +34, corresponding to the difference in mass between phenylalanine and leucine residues, whereas the signals of the N-terminus fragment ions (a_n_^+^, a_n_^*+^, [b_n_ – H]^·+^, [b_n_^*^ – H]^·+^) feature no mass shifts. Similarly, the dissociation of the phosphothreonine analogue [RGL_*p*_TYG]^·+^ (Figure S3–Supplementary) was almost identical to that of [RGL_*p*_SYG]^·+^ (Figure 2b), with their fragment ions differing by *m*/*z* 14 because of the mass difference between the phosphoserine and phosphothreonine residues; this observation again suggests similar gas-phase ion chemistry for phosphoserine- and phosphothreonine-containing peptide radical cations. Next, we compared the dissociation pathways of the radical cations of the phosphopeptides R_*p*_SYIHPF, R_*p*_TYIHPF, and R_*p*_SYIHPL and their protonated counterparts [_*p*_M + H]^+^ (Figure S4–Supplementary). As expected, the protonated phosphopeptide cations dissociate mainly through cleavages at their amide bonds, giving y_n_^+^ and b_n_^+^ ions, with predominant neutral loss of H_3_PO_4_ in the absence of any N–C_α_, C_α_–C, or side-chain bond cleavages. Comparing the CID spectra of the radical cations of phosphorylated and non-phosphorylated analogues reveals that their fragmentation behavior is generally similar, despite minor variations in the relative abundances of fragment ions and the types of low-abundance product ions. For example, CID of [RSYIHPF]^·+^ (Figure 3a) and [RGLSYG]^·+^ (Figure 3b) resulted in peptide backbone dissociation channels, including cleavage of C_α_–C, C–N, and N–C_α_ bonds along the peptide backbone (giving a_n_^+^, [b_n_ – H]^·+^, y_n_^+^, [z_n_ + H]^·+^, and [c_n_ + 2H]^+^ species) and cleavages of the side-chain C_α_–C_β_ and C_β_–C_γ_ bonds (giving [M – 106]^·+^, [M – 56]^·+^, [M – 43]^+^, [M – 29]^+^, and [M – 86]^·+^ ions, respectively); these fragmentation pathways are similar to those of the phosphorylated analogues (Figure 2a and b), except for the additional fragmentation pathways related to neutral losses of H_3_PO_4_.

### Some Factors Influencing the Neutral loss of H_3_PO_4_

**Figure 4:**
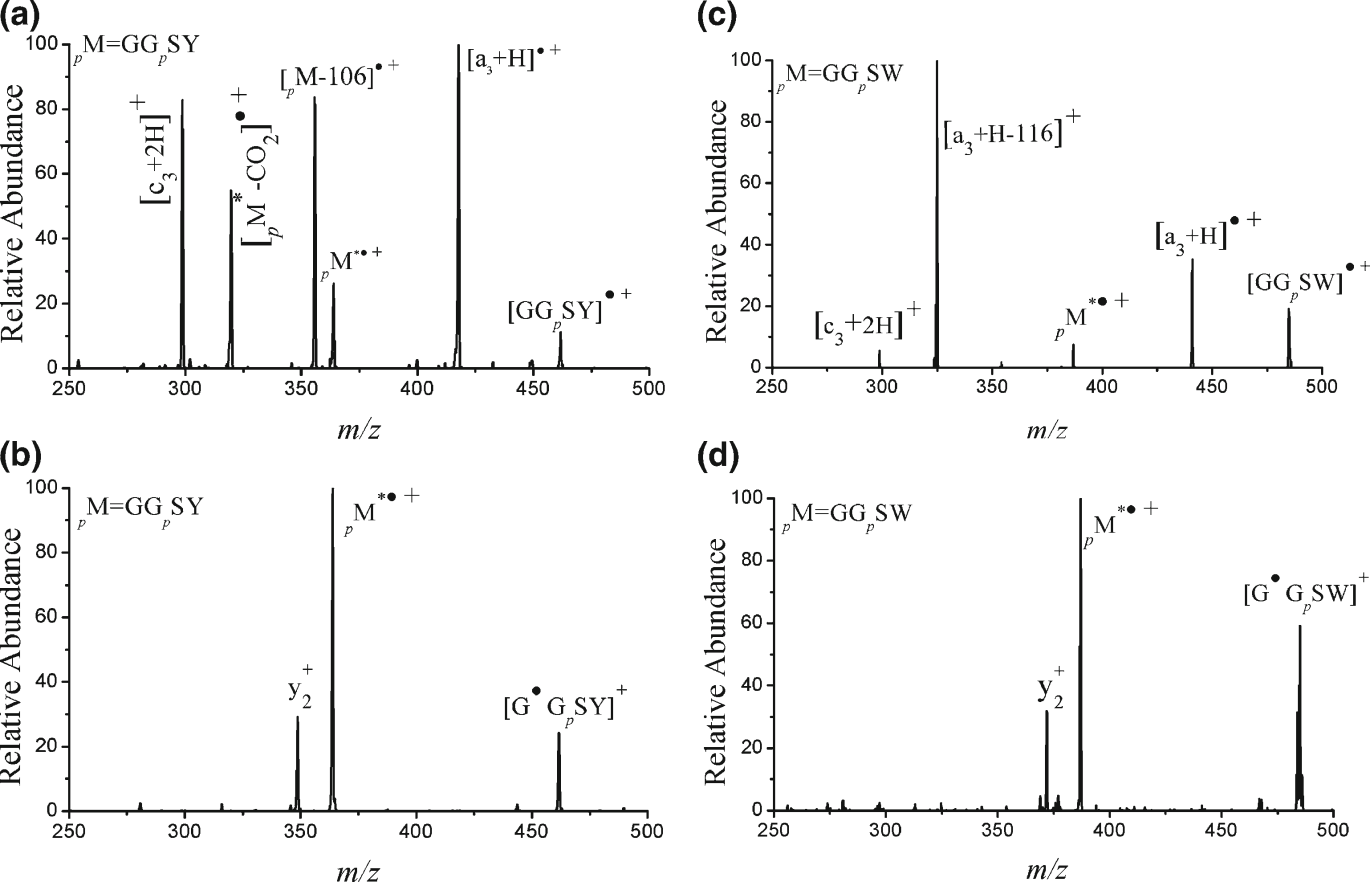
CID spectra of **(a)** [GG_*p*_SY]^·+^, **(b)** [G^·^G_*p*_SY]^+^, **(c)** [GG_*p*_SW]^·+^, and **(d**) [G^·^G_*p*_SW]^+^

The results presented above indicate that the fragmentation pathways of _*p*_M^·+^ radical cations are diverse and that they differ significantly from those of their protonated counterparts. The perception after the fact is that low-energy CID of protonated phosphopeptides can induce facile gas-phase β-elimination of H_3_PO_4_ [2]. Interestingly, the loss of H_3_PO_4_ from phosphopeptide radical cations, on the other hand, proceeds less readily in some cases, but the reasons are not obvious. The exact mechanism underlying these dissociations has yet to be determined; therefore, we further explored some of the factors governing the competition between the neutral loss of H_3_PO_4_ and other fragmentation pathways. To semiquantify H_3_PO_4_ loss, Table 1 lists the fractions of the phosphorylated peptides that underwent H_3_PO_4_ loss. The degree of neutral loss of H_3_PO_4_ depends strongly on the peptide sequence, the basicity, the site of phosphorylation, and the location of the radical along the peptide. For example, the extents of the losses of H_3_PO_4_ in the CID spectra of [GG_*p*_SY]^·+^ (Figure 4a) and [GG_*p*_SW]^·+^ (Figure 4c) were significantly different from those of the corresponding isomers with their initial radical sites well defined at the N-terminal α-carbon atom. For example, the [GG_*p*_SY]^·+^ and [GG_*p*_SW]^·+^ species produced odd-electron fragment ions [a_n_ + H]^·+^ and [c_n_ + 2H]^+^; in contrast, [G^·^G_*p*_SY]^+^ (Figure 4b) and [G^·^G_*p*_SW]^+^ (Figure 4d) predominantly formed even-electron y_n_^+^ ions and [G^·^G_*p*_SW – H_3_PO_4_]^+^ and [G^·^G_*p*_SY– H_3_PO_4_]^+^ species, respectively, with facile losses of H_3_PO_4_. Increasing the basicity of the N-terminus from arginine to glycine also increased the lability of the phosphoryl group of the phosphoserine residue, leading to more-facile H_3_PO_4_ loss. Specifically, the fractions of H_3_PO_4_ losses upon CID of [RVYIH_*p*_SF]^·+^ and [RVGIH_*p*_SY]^·+^ were 40 % and 60 %, respectively, whereas they were 19 % and 15 % for [GVYIH_*p*_SF]^·+^ and [GVGIH_*p*_SY]^·+^, respectively. The fraction of H_3_PO_4_ loss was slightly more pronounced when the phosphoserine residue was positioned next to the N-terminus.

### Theoretical Examination of the Elementary Steps Associated with H_3_PO_4_ Loss

**Figure 5:**
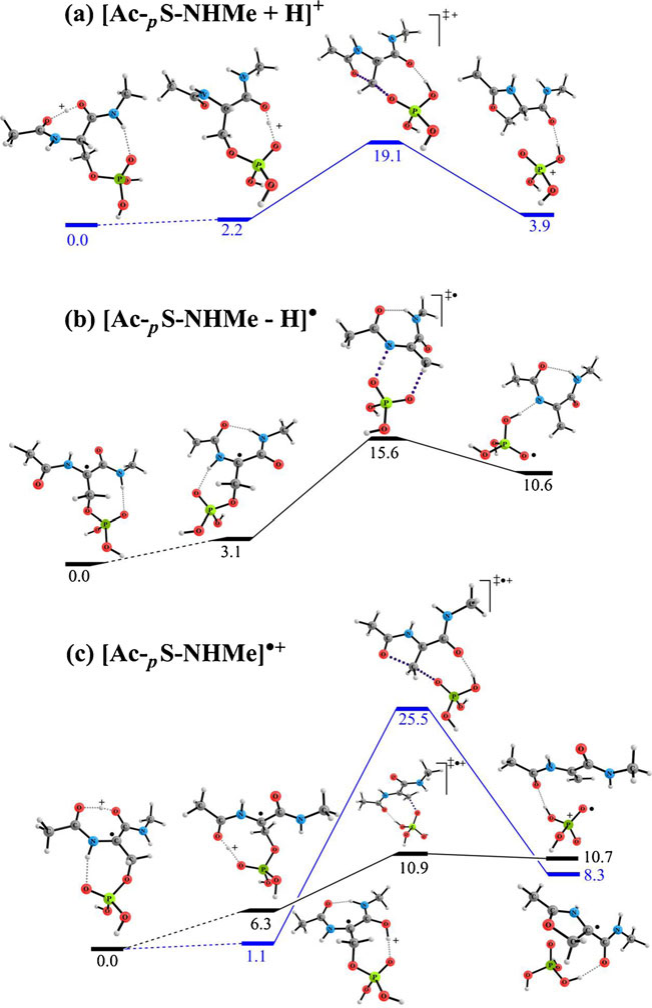
Potential energy surfaces for the H_3_PO_4_ loss of *N*-acetylphosphorylserine methylamide analogues **(a)** cation through charge-driven pathways, **(b)** radical through radical-driven pathways, and **(c)** radical cation through charge-directed (in blue) and radical-driven (in black) pathway. The relative enthalpies at 0K at B3LYP/6-311++G(d,p) level are shown in kcal mol^–1^

To gain further mechanistic insight into the losses of H_3_PO_4_ from phosphopeptide radical cations, we used *N*-acetylphosphorylserine methylamide (Ac-_*p*_S-NHMe) analogues as simple model systems to examine plausible elementary steps associated with the H_3_PO_4_ loss by means of DFT calculations at the B3LYP/6-311++G(d,p) level. Figure 5 displays the potential energy surfaces (PESs) for [Ac-_*p*_S-NHMe + H]^+^, [Ac-_*p*_S-NHMe – H]^·^ , and [Ac-_*p*_S-NHMe]^·+^, which model H_3_PO_4_ losses from a peptide that features, respectively, only the charge, only the radical, and both the charge and the radical at the phosphoserine residue.

Several mechanisms have been proposed for H_3_PO_4_ losses from protonated peptides [32], among which the most favorable involves nucleophilic attack on the β-carbon atom of the _*p*_S residue by its neighboring amide oxygen atom to form a five-membered oxazoline ring [2, 32, 33]. For our current model, we predicted the energy barrier against such charge-induced H_3_PO_4_ elimination to be 22.3 kcal mol^–1^ (Figure 5a). Radical-induced H_3_PO_4_ loss can proceed via homolytic C_β_–O bond cleavage of the _*p*_S residue that contains an α-carbon-centered radical. If no excess proton is available in the vicinity of the _*p*_S residue, we estimate the energy barrier to be 15.6 kcal mol^–1^ (Figure 5b). When an excess proton is available, as in the model as presented in Figure 5c, the energy barrier against the α-radical-induced homolytic C_β_–O bond cleavage is lowered further (to 10.9 kcal mol^–1^). The charge-induced five-membered-ring mechanism is also possible for the model in Figure 5c, with a higher energy barrier of 25.5 kcal mol^–1^. Nevertheless, the barriers for the H_3_PO_4_ losses from the currently studied model systems (10–26 kcal mol^–1^) are comparable with the values for the backbone cleavages of other tripeptide radical cations, ranging approximately from 17 to 34 kcal mol^–1^ [34–38]. Thus, both the charge and radical can play important roles in the loss of H_3_PO_4_ from molecular peptide radical cations.

## Conclusion

We have generated phosphorylated peptide radical cations through low-energy CID of corresponding ternary ligated Cu(II)– and Co(III)–peptide complexes. Our CID experiments on _*p*_M^·+^ revealed significant, yet different, extents of neutral losses of H_3_PO_4_. Dissociation of phosphorylated peptide radical cations yields not only conventional b/y ions, which are commonly observed from the dissociation of their protonated counterparts, but also [c_n_ + 2H]^+^ and [z_n_ + H]^·+^ ions, which are also sequence-informative, as well as side-chain losses. Low-energy CID of phosphopeptide radical cations generated sequences of fragment ions similar to those of their non-phosphorylated radical cationic counterparts, suggesting that phosphorylation does not significantly influence backbone fragmentation of molecular peptide radical cations, despite variations in the relative abundances of the fragment ions. The fraction of radical cations undergoing H_3_PO_4_ loss was affected by the sequence of the peptide, the site of the radical, and the basicity of the peptide. Preliminary calculations revealed that the losses of H_3_PO_4_ occurring through charge- and radical-induced mechanisms are both energetically favorable, generally featuring relatively low activation barriers.

## Electronic Supplementary Material

Below is the link to the electronic supplementary material.

ESM 1 (PDF 419 kb)

## References

[1] Manning G, Whyte DB, Martinez R, Hunter T, Sudarsanam S (2002). The protein kinase complement of the human genome. Science.

[2] Palumbo AM, Tepe JJ, Reid GE (2008). Mechanistic insights into the multistage gas-phase fragmentation behavior of phosphoserine- and phosphothreonine-containing peptides. J. Proteome Res..

[3] Ruijtenbeek R, Versluis C, Heck AJR, Redegeld FAM, Nijkamp FP, Liskamp RM (2002). Characterization of a phosphorylated peptide and peptoid and peptoid–peptide hybrids by mass spectrometry. J. Mass Spectrom..

[4] Kang J, Toita R, Jiang Y, Niidome T, Katayama Y (2006). Simultaneous analysis of phosphorylated peptides by MALDI-TOF-MS. Chromatographia.

[5] Bailey CM, Sweet SMM, Cunningham DL, Zeller M, Heath JK, Cooper HJ (2009). SLoMo: automated site localization of modifications from ETD/ECD mass spectra. J. Proteome Res..

[6] Gehrig PM, Roschitzki B, Rutishauser D, Reiland S, Schlapbach R (2009). Phosphorylated serine and threonine residues promote site-specific fragmentation of singly charged, arginine-containing peptide ions. Rapid Commun. Mass Spectrom..

[7] Hoffert JD, Knepper MA (2008). Taking aim at shotgun phosphoproteomics. Anal. Biochem..

[8] Laskin J, Futrell JH (2005). Activation of large ions in FT-ICR mass spectrometry. Mass Spectrom. Rev..

[9] Zubarev RA (2003). Reactions of polypeptide ions with electrons in the gas phase. Mass Spectrom. Rev..

[10] Syka JEP, Coon JJ, Schroeder MJ, Shabanowitz J, Hunt DF (2004). Peptide and protein sequence analysis by electron transfer dissociation mass spectrometry. Proc. Natl. Acad. Sci. U.S.A..

[11] Kemp M, Roitberg A, Mujica V, Wanta T, Ratner MA (1996). Molecular wires: extended coupling and disorder effects. J. Phys. Chem..

[12] Schlag EW, Lin SH, Weinkauf R, Rentzepis PM (1998). Dynamical principles in biological processes. Proc. Natl. Acad. Sci. U.S.A..

[13] Levis RJ (1994). Laser desorption and ejection of biomolecules from the condensed phase into the gas phase. Annu. Rev. Phys. Chem..

[14] Hodyss R, Cox HA, Beauchamp JL (2005). Bioconjugates for tunable peptide fragmentation: free radical initiated peptide sequencing (FRIPS). J. Am. Chem. Soc..

[15] Matsumoto Y, Watanabe K (2006). Coherent vibrations of adsorbates induced by femtosecond laser excitation. Chem. Rev..

[16] Ly T, Julian RR (2007). Residue-specific radical-directed dissociation of whole proteins in the gas phase. J. Am. Chem. Soc..

[17] Ly T, Julian RR (2009). Ultraviolet photodissociation: developments towards applications for mass-spectrometry-based proteomics. Angew. Chem. Int. Ed..

[18] Hopkinson, A.C., Siu, K.W.M.: Peptide Radical Cations. In *Principles of Mass Spectrometry Applied to Biomolecules*. John Wiley and Sons: New Jersey. 301–355 (2006)

[19] Hopkinson AC (2009). Radical cations of amino acids and peptides: structures and stabilities. Mass Spectrom. Rev..

[20] Barlow CK, McFadyen WD, O’Hair RAJ (2005). Formation of cationic peptide radicals by gas-phase redox reactions with trivalent chromium, manganese, iron, and cobalt complexes. J. Am. Chem. Soc..

[21] Laskin J, Yang Z, Lam C, Chu IK (2007). Charge-remote fragmentation of odd-electron peptide ions. Anal. Chem..

[22] Chu IK, Lam CNW (2005). Generation of peptide radical dications via low-energy collision-induced dissociation of [Cu^II^(terpy)(M + H)]^**·**3+^. J. Am. Soc. Mass Spectrom..

[23] Chu IK, Lam CNW, Siu SO (2005). Facile generation of tripeptide radical cations in vacuo via intramolecular electron transfer in Cu^II^ tripeptide complexes containing sterically encumbered terpyridine ligands. J. Am. Soc. Mass Spectrom..

[24] Lam CNW, Chu IK (2006). Formation of anionic peptide radicals in vacuo. J. Am. Soc. Mass Spectrom..

[25] Lam CNW, Ruan EDL, Ma CY, Chu IK (2006). Non-zwitterionic structures of aliphatic-only peptides mediated the formation and dissociation of gas phase radical cations. J. Mass Spectrom..

[26] Chu IK, Siu SO, Lam CNW, Chan JCY, Rodriquez CF (2004). Formation of molecular radical cations of aliphatic tripeptides from their complexes with Cu-II(12-crown-4). Rapid Commun. Mass Spectrom..

[27] Chan WC, White PD (2000). Fmoc solid phase peptide synthesis: A practical approach.

[28] Henke W, Kremer S, Reinen D (1983). Cu^2+^ in five-coordination: a case of a second-order Jahn–Teller effect. 1. Structure and spectroscopy of the compounds Cu(terpy)X_2_**·**nH_2_O. Inorg. Chem..

[29] Varkey SP, Ratnasamy C, Ratnasamy P (1998). Zeolite-encapsulated manganese(III)salen complexes. J. Mol. Catal. A: Chem..

[30] Frisch, M.J., Trucks, G.W., Schlegel, H.B., Scuseria, G.E., Robb, M.A., Cheeseman, J.R., Montgomery, J.A. Jr., Vreven, T., Kudin, K.N., Burant, J.C., Millam, J.M., Iyengar, S.S., Tomasi, J., Barone, V., Mennucci, B., Cossi, M., Scalmani, G., Rega, N., Petersson, G.A., Nakatsuji, H., Hada, M., Ehara, M., Toyota, K., Fukuda, R., Hasegawa, J., Ishida, M., Nakajima, T., Honda, Y., Kitao, O., Nakai, H., Klene, M., Li, X., Knox, J.E., Hratchian, H.P., Cross, J.B., Bakken, V., Adamo, C., Jaramillo, J., Gomperts, R., Stratmann, R.E., Yazyev, O., Austin, A.J., Cammi, R., Pomelli, C., Ochterski, J.W., Ayala, P.Y., Morokuma, K., Voth, G.A., Salvador, P., Dannenberg, J.J., Zakrzewski, V.G., Dapprich, S., Daniels, A.D., Strain, M.C., Farkas, O., Malick, D.K., Rabuck, A.D., Raghavachari, K., Foresman, J.B., Ortiz, J.V., Cui, Q., Baboul, A.G., Clifford, S., Cioslowski, J., Stefanov, B.B., Liu, G., Liashenko, A., Piskorz, P., Komaromi, I., Martin, R.L., Fox, D.J., Keith, T., Al-Laham, M.A., Peng, C.Y., Nanayakkara, A., Challacombe, M., Gill, P.M.W., Johnson, B., Chen, W., Wong, M.W., Gonzalez, C., Pople, J.A. *Gaussian 03, Revision C.02*. Gaussian: Wallingford CT, (2004)

[31] Chu IK, Laskin J (2011). Formation of peptide radical ions through dissociative electron transfer in ternary metal–ligand–peptide complexes. Eur. J. Mass Spectrom..

[32] Rožman M (2011). Modeling of the gas-phase phosphate group loss and rearrangement in phosphorylated peptides. J. Mass Spectrom..

[33] Palumbo AM, Smith SA, Kalcic CL, Dantus M, Stemmer PM, Reid GE (2011). Tandem mass spectrometry strategies for phosphoproteome analysis. Mass Spectrom. Rev..

[34] Paizs B, Suhai S (2005). Fragmentation pathways of protonated peptides. Mass Spectrom. Rev..

[35] Grewal RN, El Aribi H, Harrison AG, Siu KWM, Hopkinson AC (2004). Fragmentation of protonated tripeptides: the proline effect revisited. J. Phys. Chem. B.

[36] Song T, Ng DCM, Quan Q, Siu C-K, Chu IK (2011). Arginine-facilitated α- and π-radical migrations in glycylarginyltryptophan radical cations. Chem. Asian J..

[37] Song T, Ng DCM, Quan Q, Siu C-K, Chu IK (2012). Intramolecular hydrogen atom migration along the backbone of cationic and neutral radical tripeptides and subsequent radical-induced dissociations. Phys. Chem. Chem. Phys..

[38] Hao, Q., Song, T., Ng, D.C.M., Quan, Q., Siu, C.-K., Chu, I.K.: Arginine-facilitated isomerization: radical-induced dissociation of aliphatic radical cationic glycylarginyl(iso)leucine tripeptides. *J. Phys. Chem. B* (2012). doi:10.1021/jp301882p 10.1021/jp301882p 22671034

