# Supplementary material for: Formation and Dissociation of Phosphorylated Peptide Radical Cations
Source: J Am Soc Mass Spectrom. 2012 Sep 12;23(12):2094–101. doi: 10.1007/s13361-012-0479-7 (PMC3514703; doi:10.1007/s13361-012-0479-7)
Supplement: Supplementary file 1 — (PDF 419 kb) [file 13361_2012_479_MOESM1_ESM.pdf]

Supplementary Information

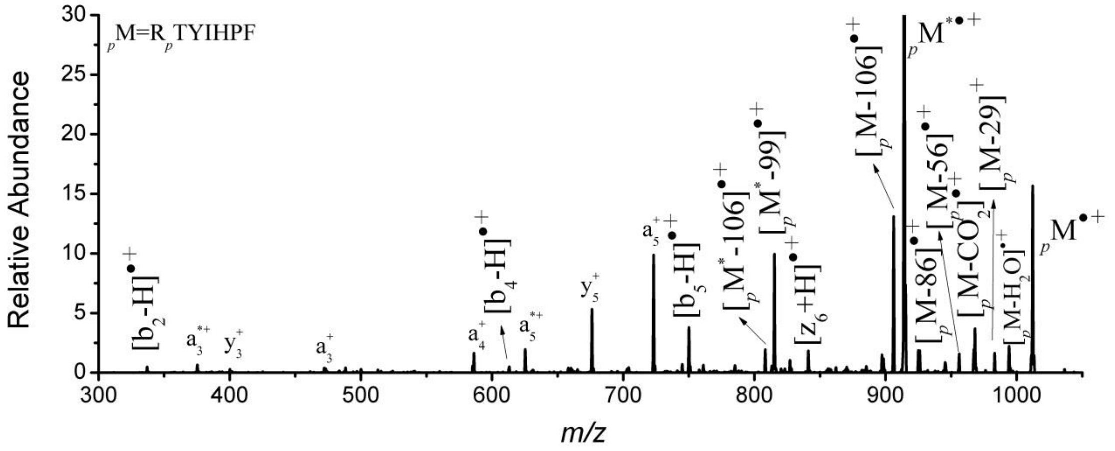

Figure S1. CID spectrum of R_pTYIHPF radical cation.

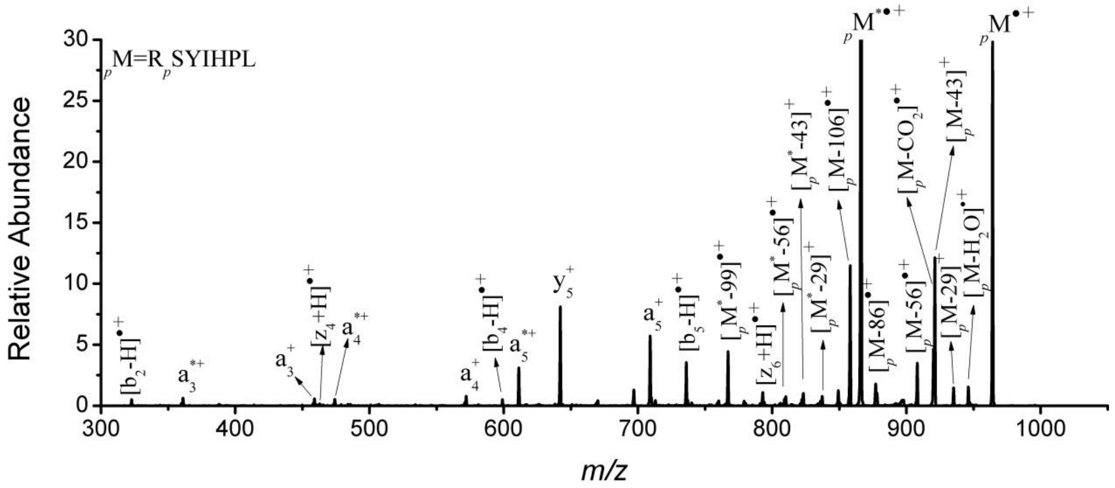

Figure S2. CID spectrum of R_pSYIHPL radical cation.

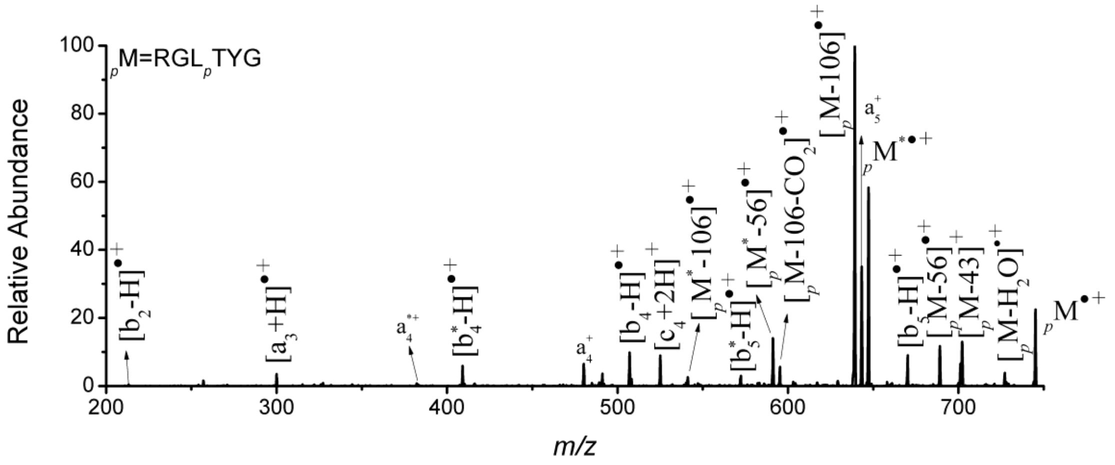

Figure S3. CID spectrum of RGL_pTYG radical cation.

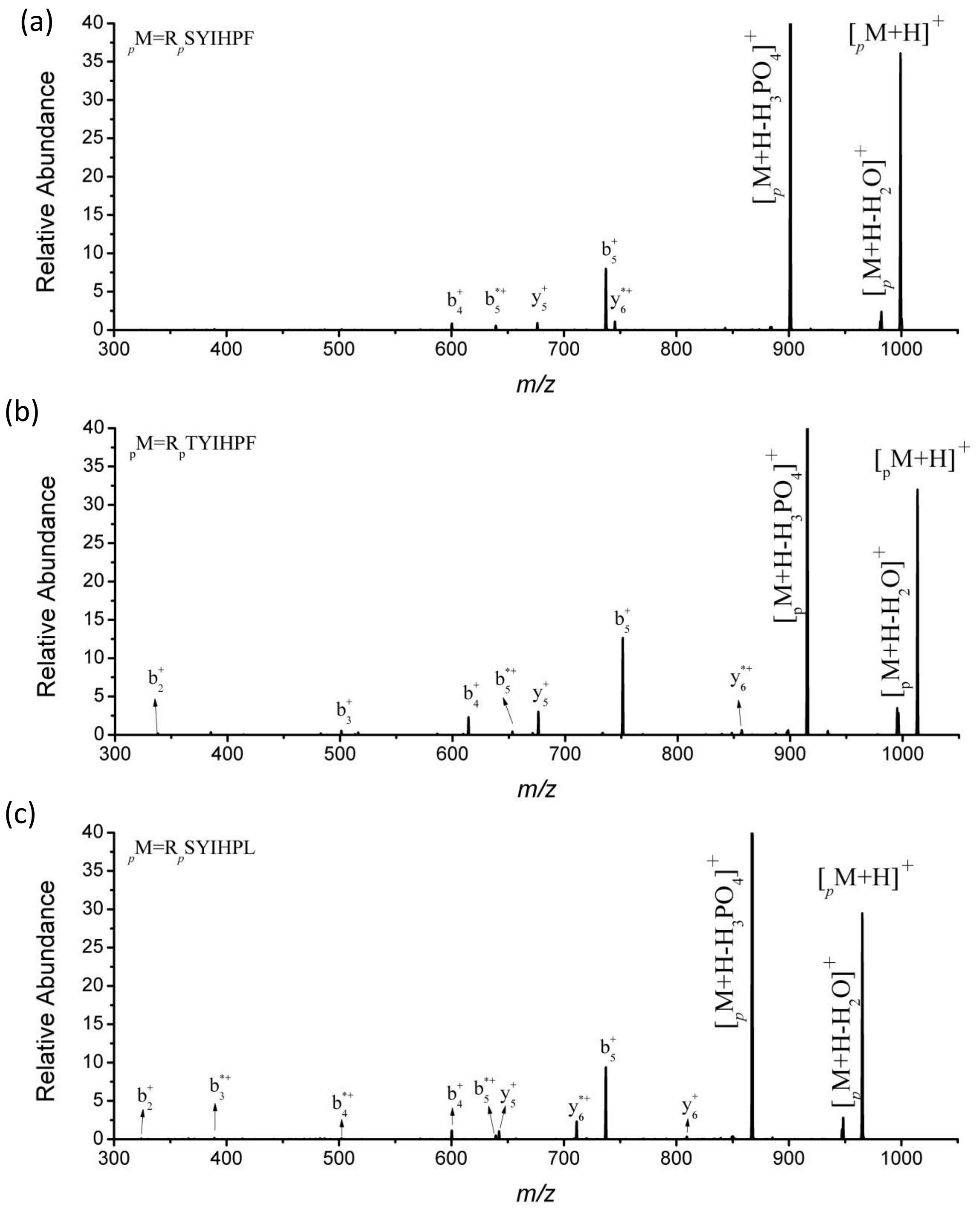

Figure S4. CID spectra of (a) protonated R$_p$SYIHPF, (b) protonated R$_p$TYIHPF, and (c) protonated R$_p$SYIHPL.

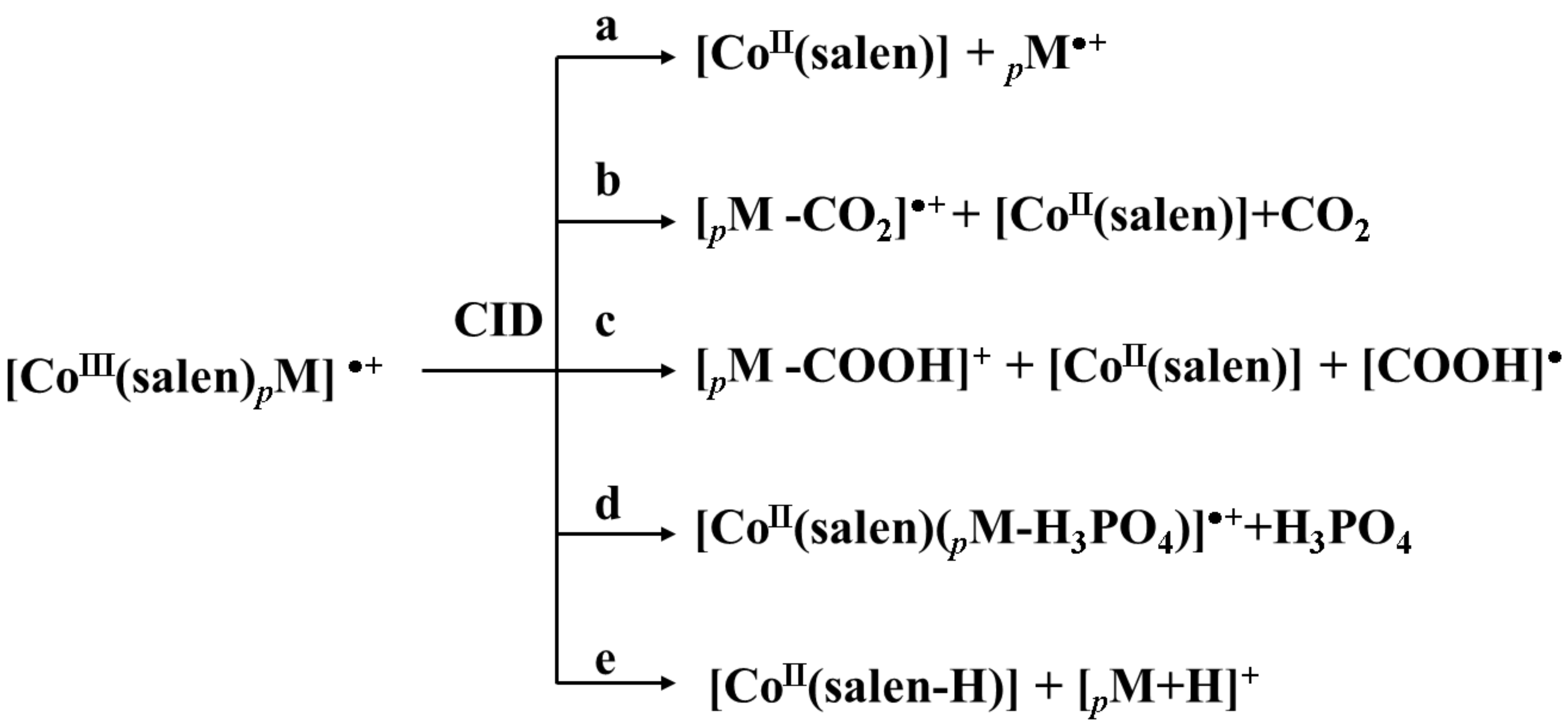

Scheme S1. Collision-induced dissociation pathway of $[Co^{III}(L)_pM]^{\bullet+}$ complex, where ${}_pM$ = phosphoserine or phosphothreonine peptide.
